# Domesticating *Vigna Stipulacea*: A Potential Legume Crop With Broad Resistance to Biotic Stresses

**DOI:** 10.3389/fpls.2019.01607

**Published:** 2019-12-06

**Authors:** Yu Takahashi, Hiroaki Sakai, Yuki Yoshitsu, Chiaki Muto, Toyoaki Anai, Muthaiyan Pandiyan, Natesan Senthil, Norihiko Tomooka, Ken Naito

**Affiliations:** ^1^Genetic Resources Center, NARO, Tsukuba, Japan; ^2^Advanced Analysis Center, NARO, Tsukuba, Japan; ^3^Kenpoku Agricultural Institute, Iwate Agricultural Research Center, Iwate, Japan; ^4^Department of Agriculture, Saga University, Saga, Japan; ^5^Agricultural College and Research Institute, Tamil Nadu Agricultural University, Thanjavur, India; ^6^Agricultural College and Research Institute, Tamil Nadu Agricultural University, Madurai, India

**Keywords:** plant domestication, wild species, legume, *Vigna*, mutant screening, seed dormancy, pod shattering, bulked segregant analysis

## Abstract

Though crossing wild relatives to modern cultivars is a usual means to introduce alleles of stress tolerance, an alternative is *de novo* domesticating wild species that are already tolerant to various kinds of stresses. As a test case, we chose *Vigna stipulacea* Kuntze, which has fast growth, short vegetative stage, and broad resistance to pests and diseases. We developed an ethyl methanesulfonate–mutagenized population and obtained three mutants with reduced seed dormancy and one with reduced pod shattering. We crossed one of the mutants of less seed dormancy to the wild type and confirmed that the phenotype was inherited in a Mendelian manner. *De novo* assembly of *V. stipulacea* genome, and the following resequencing of the F2 progenies successfully identified a Single Nucleotide Polymorphism (SNP) associated with seed dormancy. By crossing and pyramiding the mutant phenotypes, we will be able to turn *V. stipulacea* into a crop which is yet primitive but can be cultivated without pesticides.

## Introduction

To feed the growing population in the world, we have to produce more food with less input. This is a challenging issue because of global climate change, limited water resource, and acquired resistance of pests and diseases against chemicals.

To achieve this, many scientists are now focusing on harnessing genetic diversity of genebank accessions including wild crop relatives and neglected crops (McCouch et al., 2013). One of the limitations to the above described issue is genetic vulnerability of modern cultivars. They have gone through strong bottleneck and often sacrificed resilience to biotic and abiotic stresses (Pingali, 2012). On the other hand, many wild species are well-adapted to ecological niche, which is often harsh to domesticated species (Zhang et al., 2018). Thus, utilizing the adaptability of such wild or semi-wild species will be a key to sustainable agriculture.

To fully exploit the genetic diversity of wild species, “*de novo* domestication” or “redomestication” are now proposed (Fernie and Yan, 2019). Until recently, the main idea of using wild genetic resources were to cross with cultivars to introduce resistant alleles. However, cross compatibility is often limited even between a cultivar and its close relatives. In addition, adaptation to a certain environment is often a complex trait with multiple genes involved. On the other hand, domestication-related traits often arose with loss-of-function mutations in single loci (Doebley et al., 2006). Thus, it might be easier to introduce domestication-related mutations into wild species than to introduce adaptation-related alleles into crops.

Although the technologies of sequencing and editing genomes are expected to facilitate *de novo* domestication (Shapter et al., 2013; Li et al., 2018), we still believe that simple mutagenesis + forward phenotype screening could be the easiest way to do it. To apply CRISPR/Cas9 system, one has to sequence the whole genome and develop transformation system of the plant to be domesticated. In addition, genes involved in domestication-related traits are not well-catalogued except Solanaceae, Brassicaceae, and Poaceae (Østerberg et al., 2017). As such, mutagenesis followed by forward screening is currently the only practical approach to *de novo* domesticate most of the potentially useful wild plants, such as wild legumes.

Thus, in this study, we tried to domesticate *Vigna stipulacea* Kuntze by ethyl methanesulfonate (EMS) mutagenesis followed by phenotype screening. *V. stipulacea* inhabits mainly in South Asia, and has fast growth, short vegetative stage, and resistance to pests and diseases (Tomooka et al., 2014). The seeds are edible, and some local people cultivate it mainly as pasture but sometimes as food. However, less and less farmers use it because of the high labor caused by strong behavior of pod shattering (Tomooka et al., 2011). In addition, it retains seed dormancy, which needs to be reduced for uniform germination. To improve these traits, we screened and obtained mutants with reduced pod-shattering and with reduced seed dormancy. We also identified a SNP associated with seed dormancy in one of the obtained mutants by whole genome analyses.

## Materials and Methods

### Plant Materials and Growth Condition

**Table 1** summarizes the materials tested in this study. Besides *V. stipulacea*, we used two or three accessions of three domesticated species, soybean [*Glycine max* (L.) Merr.], common bean (*Phaseolus vulgaris* L.), and cowpea [*Vigna unguiculata* (L.) Walp.], to evaluate pod shattering and seed dormancy. All the materials were provided by the NARO gene bank (https://www.gene.affrc.go.jp/index_en.php). We cultivated *V. stipulacea* in a field or in a bucket with gardening soil in a greenhouse of our institute, Tsukuba, Japan (36.030577, 140.098021). We cultivated three plants of the domesticated species in a bucket with gardening soil in a greenhouse of our institute, Tsukuba, Japan.

**Table 1:** Plant materials.

Accession no.	Scientific names	Name	Origin
JP245503	*Vigna stipulacea* Kuntze	—	India
JP31043	*Glycine max* (L.) Merr.	Williams 82	America
JP28862	*G. max* (L.) Merr.	Enrei	Japan
JP239215	*Vigna unguiculata* (L.) Walp. cv-gr. Biflora E. Westphal	—	Pakistan
JP244182	*V. unguiculata* (L.) Walp. cv-gr. Unguiculata E. Westphal	—	Pakistan
JP41232	*Phaseolus vulgaris* L.	Bayo	Peru
JP41234	*P. vulgaris* L.	Red Kidney	Peru
JP41963	*P. vulgaris* L.	Frijol Azufrado	Mexico

### Development of a Mutant Population

**Figure 1:**
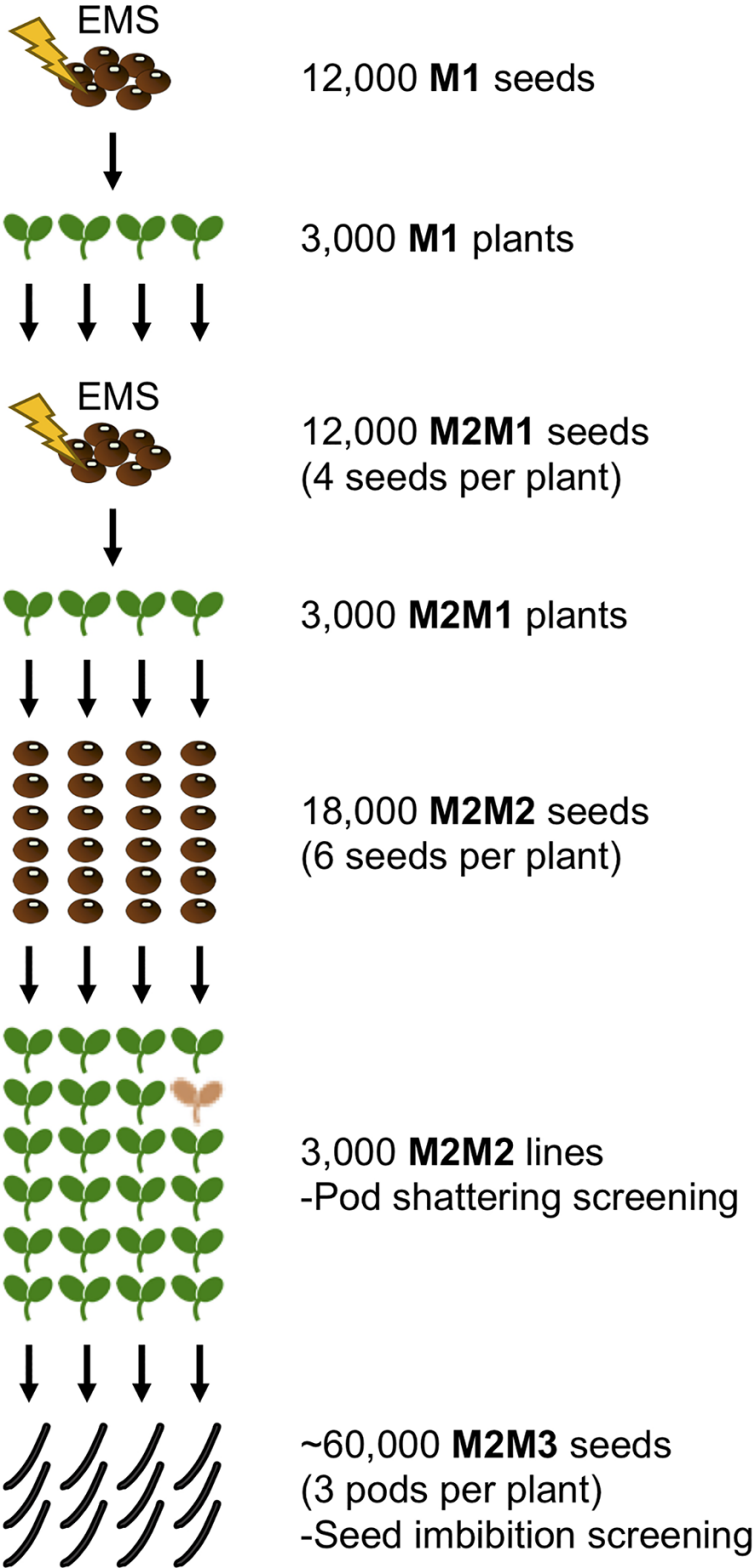
Schematic of domesticating *V. stipulacea*. After two rounds of EMS treatment, 3,000 M2M1 plants were selected to collect M2M2 seeds (six per line). The 18,000 M2M2 plants were grown in a field, where pod shattering mutants were screened for. Three pods (∼20 seeds) per plant was harvested, which were tested for seed-imbibition screening.

The process of mutant screening is shown in **Figure 1**. To increase mutation density, we adopted two cycles of successive chemical mutagenesis as described by Tsuda et al. (2015). We started with 12,000 seeds of *V. stipulacea* (JP245503). We first scratched the seed coat with a knife because it is hard and waterproof, treated the scratched seeds with 0.35% EMS solution for 8 h, and thoroughly washed them with distilled water (M1 seeds). The M1 seeds were incubated in a wet condition for 3 days and those with germination were sown. The M1 plants were cultivated in our greenhouse, where the temperature was kept above >20°C. Three pods per plant were harvested from 3,000 fertile M1 plants (M2 seeds). Of the harvested seeds, four per line (12,000 in total) were scratched and treated with 0.35% EMS solution as described above (M2M1 seeds). Germinating M2M1 seeds were sown and cultivated in a field, located in Tsukuba, Japan. Three thousand fertile plants were selected and three pods per plant were harvested (M2M2 seeds). Six seeds per M2M2 line were sown in a 1 L plastic pot filled with gardening soil and cultivated in the greenhouse until three pods per line were harvested (M2M3 seeds). Of the M2M3 lines, four lines with mutant phenotypes were selected and seven seeds per line were sown in a 7 L bucket filled with the gardening soil and cultivated in the greenhouse.

### Evaluation of Seed Dormancy and Pod Shattering

To evaluate pod shattering, we harvested 20 pods of each domesticated accession and three pods per M2M2 plant and counted the number of shattered pods. Before evaluation, harvested pods were left at room temperature for a month and then completely dried at 40°C for 24 h in an incubator.

To evaluate seed dormancy, 20 seeds were soaked in distilled water and incubated in a dark incubator at 25°C, and imbibed seeds were counted daily until the 3^rd^ day, twice a week until the 14^th^ day, and weekly until the 28^th^ day. We replicated the measurements by seven times and performed Dunnett test to compare averages of each time point between the wild type and each mutant line.

### Morphological Observation

We observed the imbibing seeds to detect the imbibition start point. We also observed pod sclerenchyma (tissue with dead cells with thickened secondary wall) with a stereoscopic microscope (ECLIPSE Ci-L, Nikon, Tokyo, Japan). Three pods were collected from each individual and were sliced by a microtome (MTH-1, Nippon Medial & Chemical Instruments Co., Ltd. Osaka, Japan), and stained with Phloroglucinol-HCL solution (1 g phloroglucin in 50 mL ethanol + 25 mL concentrated hydrochloric acid).

### Whole Genome Sequencing, Assembly, and Annotation

We sequenced the whole genome of *V. stipulacea* (JP245503) with RSII sequencer (Pacific Biosciences, Menlo Park, CA), as we have done previously for azuki bean (Sakai et al., 2015). DNA was isolated from 1 g of unexpanded leaves with CTAB method and purified with Genomic Tip 20/G (Qiagen K. K. Tokyo). The extracted DNA was sheared into 20 kb fragments using g-TUBE (Covaris, MA, USA) and converted into 20 kb SMRTbell template libraries. The library was size-selected for a lower cutoff of 10 kb with BluePippin (Sage Science, MA, USA). Sequencing was performed on the PacBio RS II using P5 polymerase binding and C3 sequencing kits with 360 min acquisition. In total, 52 SMRT cells were used to obtain ∼19.6 Gb of subreads.

In total, ∼3.3 million PacBio reads were used for *de novo* assembly with Celera Assembler 8.3rc1 (asmOvlErrorRate = 0.1, asmUtgErrorRate = 0.06, asmCgwErrorRate = 0.1, asmCnsErrorRate = 0.1, asmObtErrorRate = 0.08, utgGraphErrorRate = 0.05, utgMergeErrorRate = 0.05) (Berlin et al., 2015). About 25.2x of the longest error-corrected and trimmed reads were assembled to contigs. Redundant contigs were discarded by conducting all-to-all BLASTN searches. The non-redundant contigs were polished by PacBio subreads by using Quiver in SMRT Analysis v2.2.0 (Pacific Biosciences of California, Inc.) and then further polished by Illumina short-reads using BWA 0.7.9a (Li and Durbin, 2009), Samtools 0.1.19 (Li et al., 2009), Picard 1.94 (http://picard.sourceforge.net/), and GATK 3.3 (McKenna et al., 2010). The polished contigs were scaffolded by Reference-Assisted Chromosome Assembly (RACA) program v.0.9.1.1 (Kim et al., 2013b) using the genome sequences of *Vigna angularis* and *P. vulgaris* as the reference and outgroup species, respectively.

Repetitive sequences in the genome assembly were predicted using Censor (Kohany et al., 2006) with a composite library consisting of *de novo* created library constructed by RepeatModeler 1.0.8 (http://www.repeatmasker.org) and the MIPS Repeat Element Database ver. 9.3 (Nussbaumer et al., 2013).

*Ab initio* gene prediction was done by BRAKER version 1.6 (Hoff et al., 2016) with RNA-Seq data. Besides, gene structures were predicted by genome-guided and *de novo* RNA-Seq data assembly approaches using TopHat 2.1.0 (Kim et al., 2013a), Cufflinks 2.2.1 (Trapnell et al., 2010), Trinity 2.1.1 (Grabherr et al., 2011), and PASA pipeline 2.0.2 (Haas et al., 2003; Rhind et al., 2011). Open reading frames were predicted by Transdecoder 2.0.1 and Trinotate 2.0.2 (Bryant et al., 2017). Protein sequences of the *G. max* (Wm82.a2.v1), *P. vulgaris* (v.1.0), *Medicago truncatula* Gaertn. (Mt4.0v1), and *V. angularis* (Willd.) Ohwi & H.Ohashi (VANGULARIS_V1.A1) were downloaded from Phytozome (*G. max*, *P. vulgaris*, and *M. truncatula*) (Goodstein et al., 2012) and *Vig*GS (*V. angularis*) (Sakai et al., 2016) and mapped to the genome assembly by Exonerate 2.2.0 (Slater and Birney, 2005). The *ab initio* gene models and transcript and protein alignments were then combined by EvidenceModeler 1.1.1 (Haas et al., 2008) and the predicted gene models were updated by PASA. Gene models with extremely long introns and those merged by PASA were manually curated. BUSCO v3 (Waterhouse et al., 2017) was used to evaluate protein sequences of annotated genes.

### Bulked Segregant Analysis

We performed MutMap (Abe et al., 2012) to isolate the candidate gene for the mutant phenotype. We crossed one of the mutants of increased seed imbibition (*isi1*) to the wild type (JP245503) and obtained 280 F2 seeds. We cultivated all of them, extracted DNA from an unexpanded leaf of each F2 plants by CTAB method, and obtained F3 seeds. To evaluate seed dormancy, 20 seeds of each F3 line was soaked with distilled water for 24 h to test imbibition. DNA of 59 F2 plants with mutant phenotype (100% seed imbibition) and DNA of 221 F2 plants with wild type phenotype (0% imbibition) were pooled to make the MT pool and the WT pool, respectively, and were sequenced with Illumina HiSeq X (Illumina Co. Ltd, San Diego, USA).

The obtained sequences were mapped to the draft genome sequences with bwa-0.7.17 (Li and Durbin 2009) and formatted with samtools-1.9 (Li et al., 2009) as described in the distributed manuals. SNPs were called with bcftools-1.9, and the DP4 values were used to calculate the SNP-index. If a SNP site is responsible for the mutant phenotype, the locus must be fixed with the alternative allele [a] in the MT pool ([aa] shares 100%). On the other hand, in the WT pool, the expected ratio of the reference allele [A] and the alternative [a] is 2:1, because the WT pool [AA]:[Aa]:[aa] should be 1:2:0. Thus, at the responsible locus, the expected values of SNP-index are 1.0 in the MT pool and 0.33 in the WT pool. Thus, after calculating SNP-index, we screened all the SNP sites for those that met the criterion above.

### Genotyping by Amplicon Sequencing

We genotyped 280 F2 plants by Sanger sequencing for two SNPs whose SNP-index was 1.0 in the MutMap analysis. Two primer pairs were designed to amplify a 232 bp genomic region including 6,804,429 nt (5′-gagggaatacgaagagtttaaggtt-3′ and 5′-ttgaaaaccaggtcttttctctcta-3′) and to amplify a 243 bp genomic region including 7,009,873 nt (5′-acagagcaaaagattaaacgagaga-3′ and 5′-aaagccgcttcctagtccttac-3′) on scf0015. For Sanger sequencing, we amplified the template DNA with AmpliTaq Gold 360 Master Mix (Thermo Fisher Scientific K. K., Tokyo), performed sequencing reaction with BigDye Terminator v3.1 (Thermo Fisher Scientific K. K., Tokyo), and sequenced with ABI Genetic Analyzer 3130xl (Thermo Fisher Scientific K. K., Tokyo), according to the provider’s protocol.

## Results

### Phenotypes of Seed Dormancy and Pod Shattering in Domesticated Legumes

Though many studies have investigated seed dormancy in legume crops, there are still some arguments on the sites of water entry (Smýkal et al., 2014). Thus, we evaluated seed imbibition and pod shattering in soybean, common bean, and cowpea.

**Figure 2:**
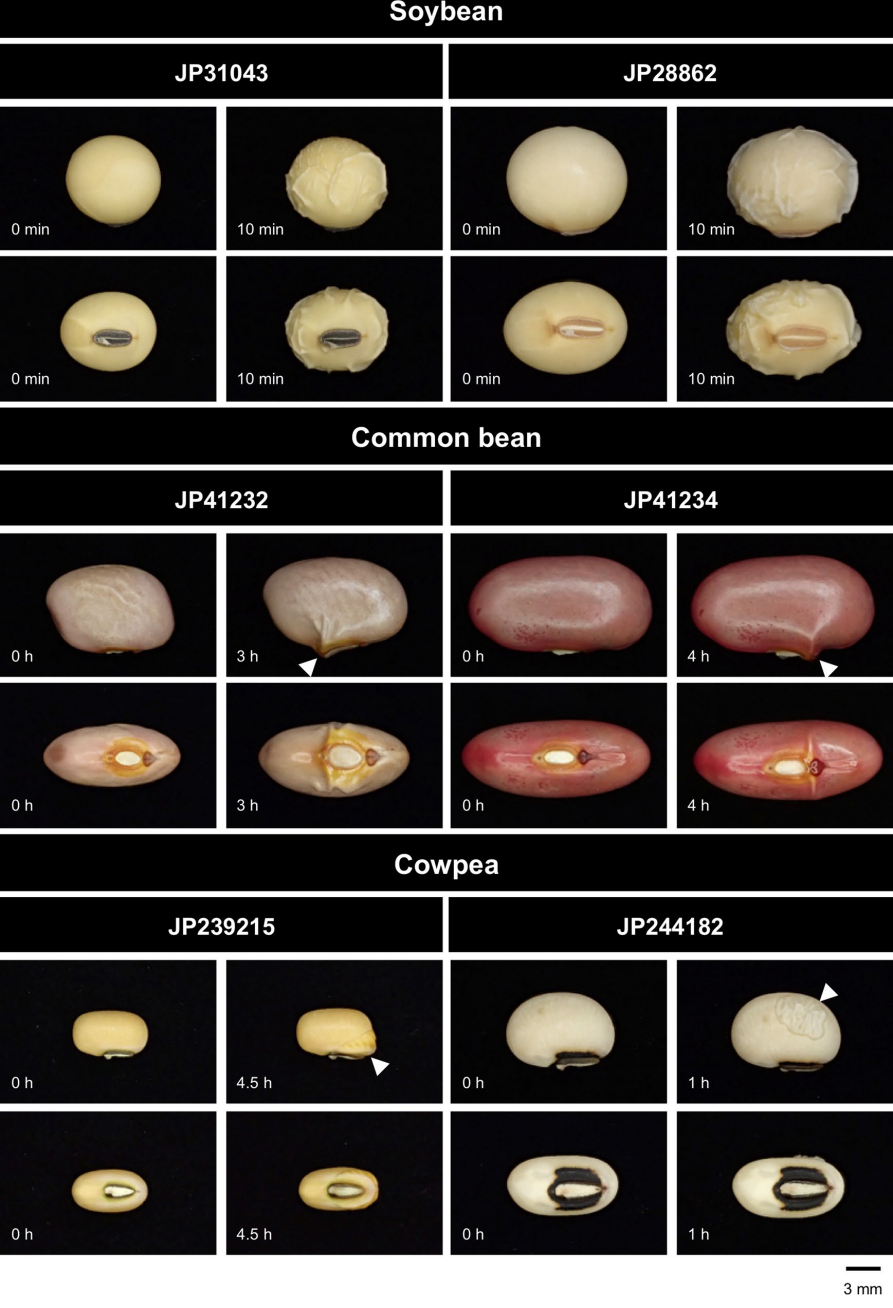
Water entry sites in domesticated legumes. The entry sites (indicated by arrowheads) varied across accessions, but did not vary within accessions.

As a result, domesticated species showed variation in both imbibition start sites and time to imbibe (**Figure 2**). Among the domesticated accessions tested, soybean imbibed within a few minutes and was the quickest. In soybean, the whole testa seemed permeable. The second quickest was the cowpea “JP244182,” where the testa was partially permeable and imbibition was observed in an hour. The common bean “JP41232” showed imbibition in 2 to 3 h, which started at the site of micropyle. It took 4 to 5 h for the common bean “JP41234” and the cowpea “JP239215,” where imbibition started at the site of lens. All the seeds were fully imbibed within 48 h.

**Figure 3:**
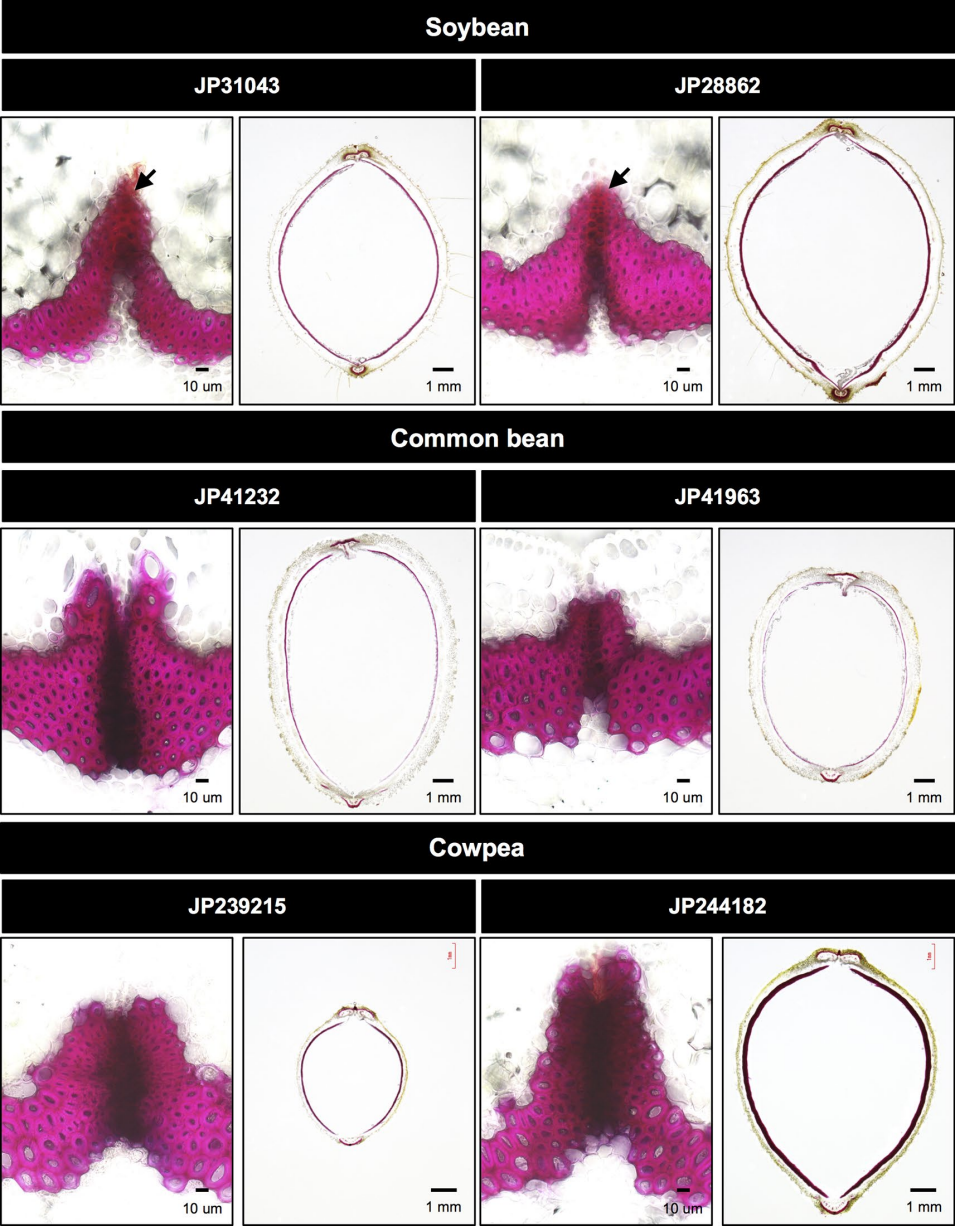
Cross-sections of seed pods in domesticated accessions. The left panel of each accession is a close-up of abscission zone. Phloroglucinol-HCl stains pod sclerenchyma with bright red while it stains abscission zone with dark red. Soybean accessions have brightly-stained sites at the tip of fiber cap cells (arrows), which indicates abscission layer is not completely formed.

We also observed abscission layers between the valves of seed pods and the sclerenchyma on the endocarp, because pod shattering is dependent on these tissues (Murgia et al., 2017). In soybean, as described by Dong et al. (2014), abscission layer was not completely formed at the fiber cap cells (**Figure 3**). Abscission layer was even less completely formed in the common bean accession JP41963 whereas it was fully formed in all other accessions (**Figure 3**). Pod sclerenchyma was thicker in the soybean accessions and the cowpea accession JP244182, whereas it was thinner in common bean accessions (**Figure 3**).

### Mutant Screening

**Figure 4:**
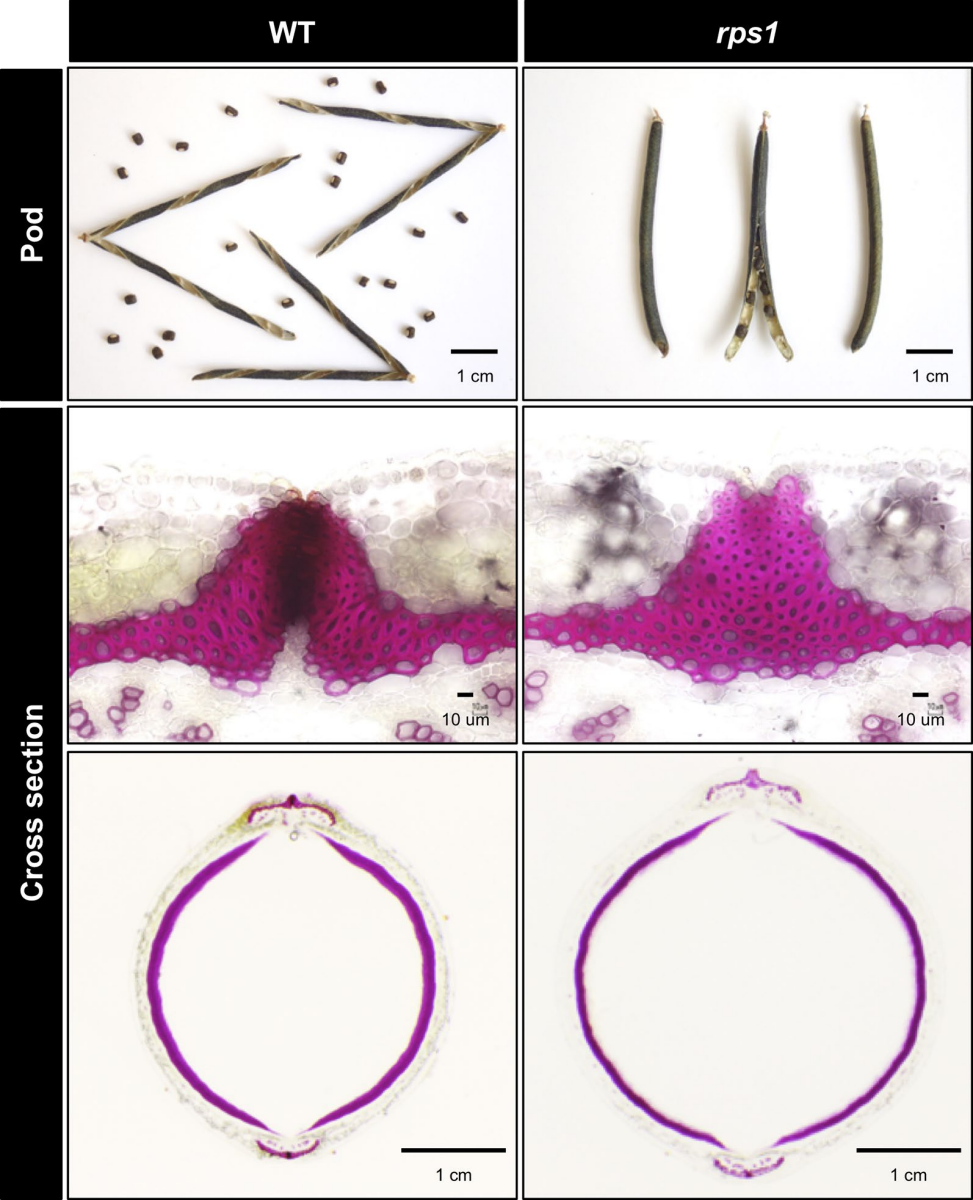
Non-shattering mutant of *V. stipulacea*. Pod shattering was strongly suppressed in the *rps1* mutant, where the abscission layer between the valves were not formed at all, whereas the thickness of sclerenchyma did not greatly change.

**Figure 5:**
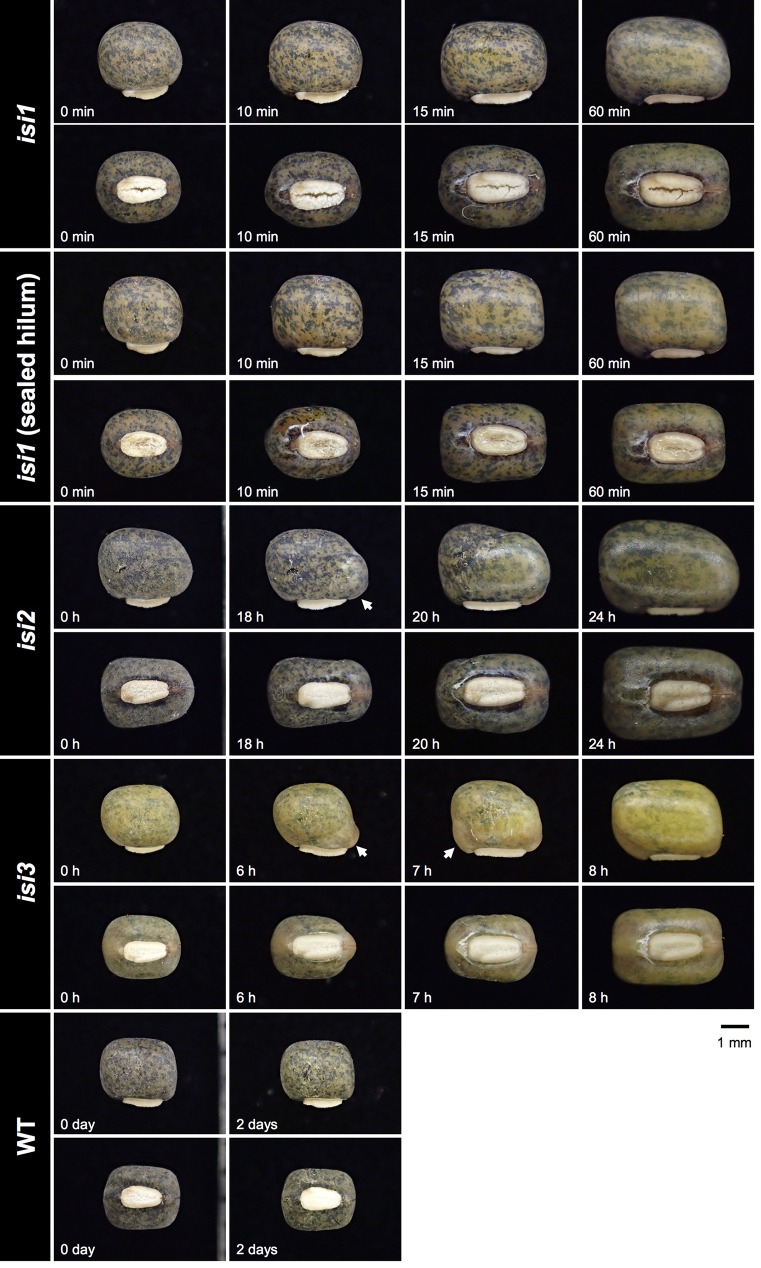
Increased seed imbibition mutants of *V. stipulacea*. Time after watering is indicated at the bottom-left of each photo. Arrows indicate where water entry was initiated.

To obtain mutants with reduced seed dormancy and reduced pod shattering, we treated 12,000 seeds of *V. stipulacea* with EMS and developed 3,000 M2M2 plants. Of the 3,000, we found one line which exhibited reduced pod shattering and designated it as *reduced pod shattering1* (*rps1*) (**Figure 4**). We also screened M2M3 seeds for seed imbibition and obtained three lines which exhibited increased seed imbibition and designated them as *increased seed imbibition1* (*isi1*), *increased seed imbibition2* (*isi2*), and *increased seed imbibition3* (*isi3*), respectively (**Figure 5**). Of them, the *isi1* seeds had cracks in the hilum, and the *isi3* seeds showed reduced pigmentation in the seed coat (**Figure 5**).

The mode of imbibition also varied across the mutants (**Figure 5**). In the *isi1*, the water entry was not necessarily through the cracks in the hilum but through the whole seed coat. Even though we sealed the hilum with glue, imbibition was quickly initiated [see the “*isi1* (sealed hilum)” in **Figure 5**]. In *isi2*, the imbibition was initiated at the lens. In *isi3*, the imbibition started near the hilum, but not necessarily through the micropyle or the lens.

### Mutant Phenotypes Regarding Seed Dormancy

**Figure 6:**
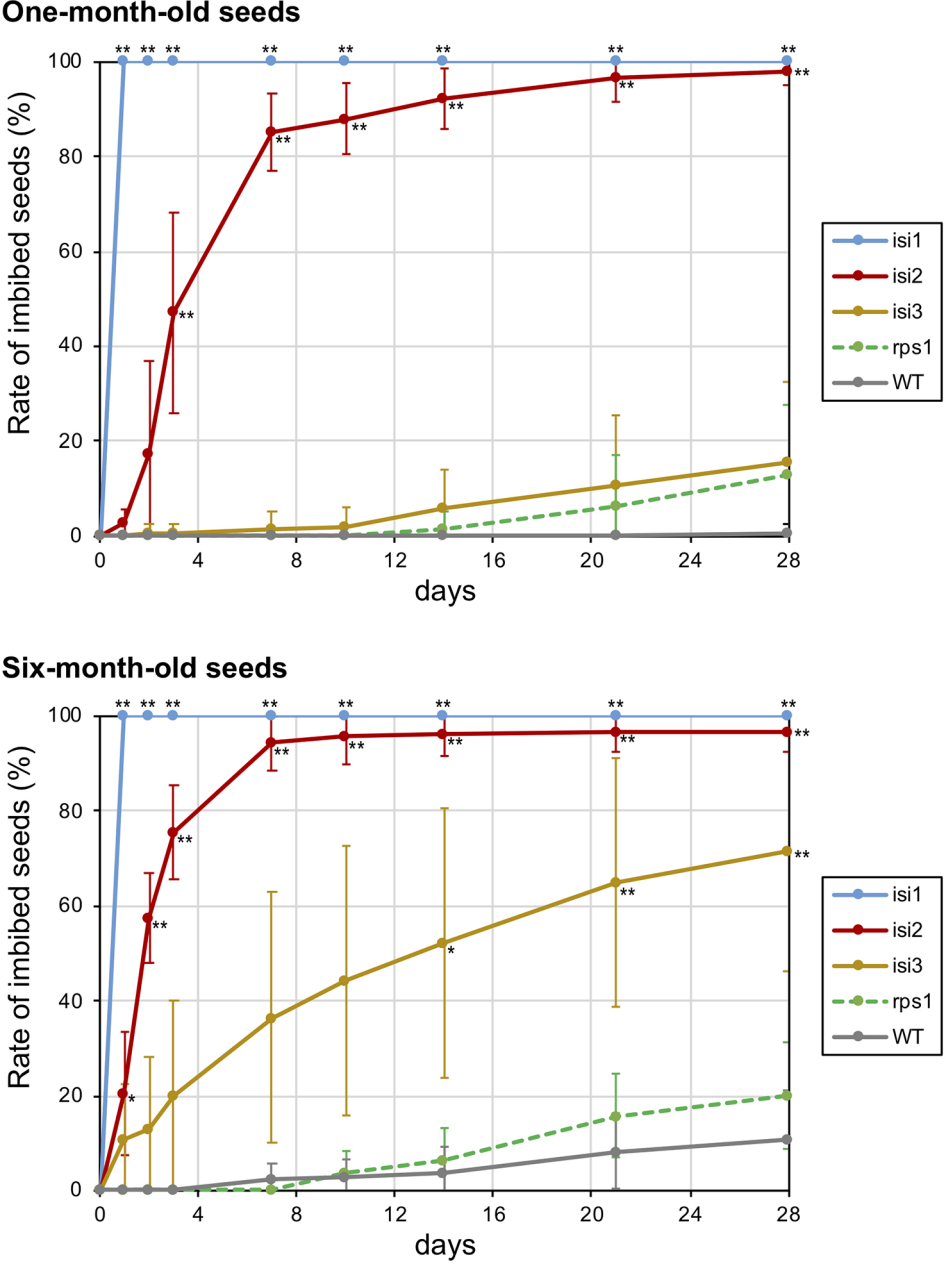
Rate of imbibed seeds in the mutant plants over time. Twenty seeds of 1 and 6 months old were soaked in distilled water and number of imbibed seeds were manually counted twice a week for 4 weeks. The error bars indicate standard deviation of replicated evaluations (n = 7). Asterisks indicate that the mean values are significantly different from the wild type (** for *p* < 0.01 and * for *p* < 0.05).

To elucidate the extent of seed dormancy in the mutant lines, we put the seeds on wet filter paper and evaluated the rate of imbibed seeds (**Figure 6**). We also tested the seeds of 1 and 6 months old to elucidate the effect of duration time after harvesting. When we did the experiment with the wild type seeds, the imbibition rate was zero for at least 4 weeks in 1-month-old seeds and was 11% in the 6-month-old seeds (**Figure 6**).

When we tested the mutant seeds of 1 month old, the imbibition was quick in *isi1* and *isi2*, whereas it was slow in *isi3*. In *isi1*, all the seeds fully imbibed within 1 h (**Figure 6**). In *isi2*, 85% of the seeds imbibed within a week, and almost 100% did so within 4 weeks. In contrast, the imbibition rate of the *isi3* seeds was only 16% in 4 weeks, which was not significantly different from the wild type. The imbibition rate of the reduced shattering mutant *rps1* was also higher but not significantly different compared to the wild type (**Figure 6**).

When we tested the mutant seeds of 6 months old, the overall imbibition rate was increased compared to the experiment with 1-month-old seeds (**Figure 6**). In *isi1*, the seeds also fully imbibed within 1 h. In *isi2*, the imbibition rate was already significantly different from the wild type in 1 day, more than 90% of the seeds imbibed within a week, and reached plateau (∼95%) in two weeks. In *isi3*, 20% of the seeds imbibed in 3 days, 36% in a week, and 71% in 4 weeks. However, this line exhibited a large standard deviation between the replicates and was not significantly different before 2 weeks (**Figure 6**). In *rps1*, ∼20% of the seeds imbibed in 4 weeks, which was twice as high as the wild type but not significantly different from the wild type was not significant.

### Mutant Phenotypes Regarding Pod Shattering

To evaluate pod shattering in the mutant lines, we calculated the rate of shattering of the harvested pods which were completely dried in the incubator. Whereas the shattering rate was 100% in the wild type, it was 0% in the *rps1* mutant (**Table 2**). The *rps1* mutant also showed a reduced twisting of the seed pod. The number of twists/cm in the pods was 0.371 ± 0.018 in the *rps1* mutant, which was less than half of the wild type (0.866 ± 0.022) (**Table 2**).

**Table 2:** Phenotypic data of mutant lines.

Names	Symbol	No. twist/cm ± SD	Pod shattering rate (%) ± SD
*increased seed imbibition 1*	*isi1*	0.579 ± 0.093	73.99 ± 18.53
*increased seed imbibition 2*	*isi2*	0.615 ± 0.149	100 ± 0
*increased seed imbibition 3*	*isi3*	0.712 ± 0.066	100 ± 0
*reduced pod shattering 1*	*rps1*	0.371 ± 0.018	0 ± 0
Wild type (JP245503)	WT	0.866 ± 0.022	100 ± 0

Interestingly, the *isi1*, one of the mutants of seed imbibition, also exhibited slightly reduced shattering rate (73.99 ± 18.53%) and number of twists/cm (0.579 ± 0.093) (**Table 2**). Other mutants were also slightly reduced in number of twists/cm, but their shattering rate was 100% (**Table 2**).

We also observed cross-sections of seed pods and found the *rps1* mutant did not form abscission layer between the valves at all (**Figure 4**).

### Whole Genome Sequence and Annotation of *V. stipulacea*

To build a reference sequence of *V. stipulacea,* we sequenced the genomic DNA with a PacBio sequencer, assembled, and annotated. We obtained 19.6 Gbp of subreads using 52 SMRT cells. Genome size of the *V. stipulacea* was estimated to be ∼445.1 Mbp based on the k-mer frequency distribution obtained from 10.3 Gbp of the Illumina short-reads. The assembled contigs showed N50 length of ∼1.9 Mbp and mean length of ∼169.8 kbp and covered ∼387.7 Mbp (87.9%) of the estimated genome size (**Table 3**). Out of the 2,102 scaffolds, 52 scaffolds were comprised of 233 contigs scaffolded by RACA.

**Table 3:** Stats of the assembled genome sequence of *V. stipulacea*.

Statistic items	Stats
Estimated genome size (Mbp)	441.5
Total contig length (bp)	387,726,606
No. contigs	2,283
N50 contig (bp)	1,936,224
Mean contig length (bp)	169,832
Total scaffold length (bp)	387,906,601
No. total scaffolds	2,102
N50 scaffold (bp)	8,789,545
	
No. protein coding genes	26,038
Mean protein length	419.6
Complete BUSCOs	1,379 (95.8%)
Complete and single-copy BUSCOs	1,274 (88.5%)
Complete and duplicated BUSCOs	105 (7.3%)
Fragmented BUSCOs	14 (1.0%)
Missing BUSCOs	47 (3.2%)

By combining the *ab initio* gene models and transcript and protein alignments, 26,038 protein coding genes were predicted on the assembled genome (**Table 3**). We assessed the completeness of the gene set by BUSCO and found that 95.8% of the BUSCO gene models were completely detected (**Table 3**). The BUSCO metrics was almost the same as that assessed for the recently published high quality genome sequence of cowpea (Lonardi et al., 2019), suggesting that our genome assembly covered nearly complete gene space of the *V. stipulacea* genome.

### Mapping a Locus for *isi1* Phenotype

To identify the responsible genes for the mutant phenotype, we performed MutMap analysis. Of the 280 F2 plants derived from *isi1* x WT, 221 showed the wild type phenotype and 59 showed the mutant phenotype, which was close to the expected 3:1 ratio (*p* = 0.13). We sequenced the genomic DNA of the wild type plants (WT pool) and the mutants (MT pool) and identified 33,936 SNPs across the whole genome. We then calculated SNP-index of each SNP and screened for those where it was 1.0 in MT pool and 0.3 ± 0.1 in WT pool.

As a result, we found only two on scf0015, a C to T substitution at 6,804,429 nt and a G to A at 7,009,873 nt. The C to T SNP was present in the 8^th^ exon of Vigst.0015s042600.01, which encoded CELLULOSE SYNTHASES A 7 (CesA7) protein, and turned the Glu codon into a STOP codon. The G to A SNP was present in Vigst.0015s044500.01, which encoded a nucleoporin-like protein, but was synonymous.

For further mapping, we looked for recombinants between the two candidate SNPs by direct sequencing of the SNP-containing regions in all the F2 plants. As a result, one F2 plant, which showed the wild type phenotype, was heterozygous at Vigst.0015s042600.01 but was fixed with the mutant A allele at Vigst.0015s044500.01 (see #130 in **Supplementary Table 1**). Another F2 plant, which showed the mutant phenotype, was fixed with the alternative T allele at Vigst.0015s042600.01 but was heterozygous at Vigst.0015s044500.01 (see #251 in **Supplementary Table 1**). The result was further confirmed by genotyping and phenotyping F3 plants derived from the F2 plants with recombination between the two candidate SNPs (#130, #251, and #275 in **Supplementary Table 1**). As shown in **Supplementary Table 2**, the phenotype was completely linked with the SNP at scf0015_6804429 on CesA7 gene but not with the other.

## Discussion

In this study we demonstrated a practical approach to *de novo* domesticate a wild legume species *V. stipulacea* by chemically-induced mutations. We successfully obtained three independent mutations in seed dormancy and one in pod shattering. In addition, we identified a SNP in the candidate gene, which encoded CELLULOSE SYNTHASE A 7, in one of the three mutants of reduced seed dormancy.

We demonstrated that forward screening following mutagenesis was practical enough to screen for mutants in domestication-related traits. This could be easily predicted from the fact that legume crops have reduced seed dormancy and pod shattering in various manners (**Figures 2** and **3**). This indicates that many genetic loci are involved in these traits, and loss-of-function mutation in any of them could cause such phenotypes. This is why we identified three mutants for seed dormancy and one for pod shattering from only 3,000 M2M2 lines (18,000 plants) (**Figure 1**). We also note that we obtained the mutants within 3 years since we initiated the first mutagenesis.

Of the three mutants for seed dormancy, *isi1* showed the severest phenotype and imbibition was always completed within hours (**Figures 5** and **6**). This was because of water-permeability in the seed coat, which were probably caused by the loss-of-function mutation in CesA7. CesA7 is involved in cellulose synthesis and its malfunction might have disrupted development of hilum and seed coat. The phenotype of cracked hilum had not been observed in other legume crops and makes the seed almost completely non-dormant. However, the seeds might not be suitable for long-term storage, because the cotyledons are exposed to the air and could be easily oxidized. In addition, *isi1* showed slightly lower rate of pod shattering. This could be a pleiotropic effect of the mutation, because cellulose fibers are also important for pod shattering (Suanum et al., 2016). However, we cannot exclude the possibility that *isi1* contains other mutations involved in pod shattering.

Compared to *isi1*, *isi2*, and *isi3* showed milder phenotypes (**Figure 6**). Because *isi2* seeds imbibe through lens, the mutation disturbed the same pathway as in the common bean “JP41234” or the cowpea “JP239215” (**Figure 2**). However, it took much longer time for *isi2* to complete imbibition than for those cultivars. This might be because the domesticated accessions have accumulated multiple mutations involved in seed coat permeability (Jang et al., 2015; Sun et al., 2015). As for *isi3*, the seed coat color was retarded but imbibition did not always initiate in the seed coat. It sometimes did through the lens and sometimes through the micropyle. Given the water entry sites are basically stable in the cultivated accessions, the *isi3* mutant might be a novel phenotype of seed dormancy. Or, it is also possible that *isi3* have multiple mutations involved in seed imbibition, given we are currently aware of only the seed color in this mutant. If so, such mutant alleles (except the one involved in seed pigmentation) could be segregating and lead to variation of water entry sites and imbibition rate within the mutant line (**Figure 6**). Though *isi2* and *isi3* have only partial effect on seed imbibition, double-mutant of both might be able to complete imbibition within a week with smaller effect on long-term storability.

The *rps1* mutant almost completely lost the pod shattering behavior because of suppressed formation of the abscission layer between the valves (**Figure 4**). This phenotype was similar to the effect of the domestication-type allele of SHAT1-5 in soybean (**Figure 2**) (Dong et al., 2014). Thus, the mutation in *rps1* might be in a gene involved in the SHAT1-5 pathway. The responsible gene for *rps1* phenotype might be useful for improving shattering problem in other legumes because *rps1* phenotype was severer than soybean SHAT1-5. On the other hand, however, severe disruption in development of abscission layer could increase labor to thresh. In addition, though not significant, we repeatedly observed that the *rps1* mutant exhibited slightly increased seed imbibition compared to the wild type (**Figure 6**). Such pleiotropy, unless it has other mutations involved in seed dormancy, might be because secondary wall thickening plays important roles in shattering behavior in seed pod (Suanum et al., 2016; Parker et al., 2019; Rau et al., 2019; Takahashi et al., 2019) and water permeability in seed coat (Smýkal et al., 2014; Hradilová et al., 2019).

Last but not least, the technology of long-read sequencing (Eid et al., 2009) enabled us to obtain high-quality genome assembly of *V. stipulacea*. Although we did not use further scaffolding techniques such as optical mapping (Lam et al., 2012) or Hi-C (Burton et al., 2013), it achieved the N50 of nearly 2 Mbp and the BUSCO score of 96.8 (**Table 3**). The high-quality reference genome should be the reason for that we successfully identified the candidate SNP by a single resequencing analysis.

As described above, here we report that we have achieved the first step of domesticating the wild legume *V. stipulacea*. As reported in Tomooka et al. (2011), this legume showed resistance to broad range of pests and diseases in Tamil Nadu, India, and does not need pesticides to spray. Broad range of pests and diseases resistance of *V. stipulacea* was confirmed during the mutant screening procedure conducted in our experimental field in Tsukuba, Japan. Thus, if we cross and pyramid the mutant phenotypes obtained in this study, we will be able to make *V. stipulacea* easier to cultivate and easier to harvest, and we believe it will be a step forward to accomplish low input agriculture. By screening for more useful traits from more mutagenized population, we hope this kind of “domesticated wild plants” will be popular and prevalent.

## Data Availability Statement

The raw and assembled sequence data (DRA009127) are all available from DNA Data Bank of Japan (https://www.ddbj.nig.ac.jp/dra/index.html) or *Vig*GS (https://viggs.dna.affrc.go.jp/).

## Author Contributions

NT and KN provided the idea of the study. YT, MP, NS, NT and KN planned the study. YT, CM, TA, NT and KN cultivated plants and collected data. YT, YY, HS and KN analyzed data. YT, HS, MP, NS, NT and KN wrote the paper.

## Funding

This study was supported by JSPS KAKENHI Grant Number 13J09808, 19KT0016, and 26850006. It was also partially supported by Research Supporting Program of the Advanced Analysis Center, National Agriculture and Food Research Organization (NARO) and the Genebank Project, NARO.

## Conflict of Interest

The authors declare that the research was conducted in the absence of any commercial or financial relationships that could be construed as a potential conflict of interest.
